# An exploratory cross-sectional study on the impact of education on perception of stigma by Chinese patients with schizophrenia

**DOI:** 10.1186/s12913-016-1424-4

**Published:** 2016-06-30

**Authors:** Zhibin Ren, Heqiu Wang, Bin Feng, Chenyu Gu, Yongchun Ma, Hong Chen, Bingling Li, Lanying Liu

**Affiliations:** Department of Psychiatry, Tongde Hospital, Zhejiang Chinese Medical University, 234 Gucui Road, Hangzhou, Zhejiang 310012 China

**Keywords:** Schizophrenia, Stigma, Brief psychosis rating scale (BPRS), The positive and negative syndrome scale (PANSS), The clinical global impression-severity of illness (CGI-SI) scale

## Abstract

**Background:**

Stigma is a major issue across various society and cultures, and few studies focus on the perception of stigma by Chinese patients with schizophrenia. In the current cross-sectional study, we sought to assess the extent of internalized stigma among outpatients with schizophrenia in China and to investigate whether education level correlated with the experience of stigma.

**Methods:**

Outpatients with schizophrenia were evaluated using the brief psychosis rating scale (BPRS), the positive and negative syndrome scale (PANSS), the clinical global impression-severity of illness (CGI-SI) scale and the Stigma Scale for Mental Illness (SSMI 2C). Patients were categorized into the high education and low education group according to their educational levels.

**Results:**

One hundred thirty-three subjects were included in the study. Their mean course of illness was 4.32 ± 6.14 years (range, 1 month to 15 years). Their mean BPRS score was 19.87 ± 5.46, their mean PANSS score was 44.11 ± 13.1, and their mean CGI-SI score was 2.22 ± 0.81. In addition, the mean SSMI 2C score of the high education group (7.15 ± 0.98) was markedly higher than that of the low education group (5.75 ± 0.79, *P* < 0.05). The mean domain I score of the high education group (2.30 ± 0.76) was comparable to that of the low education group (2.07 ± 0.78, *P* > 0.05). The mean domain II score of the high education group (2.42 ± 0.96) was markedly higher than that of the low education group (2.01 ± 0.79, *P* < 0.05). Moreover, the mean domain III score of the high education group (2.43 ± 0.79) was significantly higher than that of the low education group (1.67 ± 0.77, *P* < 0.05).

**Conclusions:**

Education level impacts on the perception of stigma by patients with schizophrenia and more psycho-education should be done to improve patients’ knowledge about schizophrenia.

## Background

Schizophrenia is a chronic illness that carries a heavy burden for the society and the family and individual patients. Stigma is well established as added burden for people with schizophrenia [1]. Stigma is a major problem across different societies, but the particular manifestations of stigma may vary due to apparent or subtle differences in socially or culturally accepted norms of behavior imposed on individual patients. Currently, two major types of stigma are recognized: public or social and personal stigma. Personal stigma can be 1) perceived stigma, which is what an individual patient thinks society’s beliefs are about the stigmatized patient, 2) experienced stigma, which is actual discrimination an individual patient has experienced, and 3) self-stigma: a product of the internalization of public stigma. In internalized or self-stigma, an individual patient gradually assimilates public stereotypes of mental illness to such a degree that the patient progressively loses his or her perception of himself or herself, which ultimately leads to changes in his or her behavior in a way that are consistent with the internalized perceptions [2]. Thorough investigation of the extent and correlates of internalized stigma is essential to planning for recovery programs for patients with schizophrenia.

Stigma toward patients with schizophrenia in Chinese societies is pervasive, frequently resulting in internalization of these negative conceptions and loss of self-esteem. Chen *et al.* [3] interviewed the family members of 72 patients with severe mental illness using the Family Stigma Interview (FSI) and found stigma was pervasive in the family members, especially if their children had higher levels of education. In 2005, Gao *et al.* [4] surveyed 225 convalescent patients with schizophrenia and their family members at 3 specialty hospitals in Beijing. They found that 42 % of the patients experienced unfair treatment at jobs and 56 % of their family members hid the illness from others to avoid discrimination. Huang *et al.* at Shanghai Changning District Mental Hospital [5] studied 209 patients with mental illness who were hospitalized for >12 months and found that stigma was pervasive. Phillips *et al.* [6] interviewed 1491 patients with schizophrenia in 5 mental illness institutions between 1990 and 2000 using the Camberwell Family Interview (CFI), 60 % of the patients and their family members felt moderate impact on their life because of stigma.

There has been growing interest in stigma in patients with schizophrenia including the etiology of stigma, the self-perception of patients with schizophrenia and impact of stigma on patients with schizophrenia and their family. The studies on outpatients with schizophrenia by Ritshera [7] and Sartorius [8] found that stigma was present in these patients during convalescence. Prince [9] also found that a majority (73.2 %) of patients mental illness had self-derogation and felt being discriminated against. Ritshera [7] followed up 82 outpatients with mental illnesses for 4 months and found one third of the patients had high levels of self-stigma, which may meet the criteria for depression and 2/5 of the patients reported social regression while only one fourth of the patients exhibited high level stigma resistance. Schulze *et al.* [10] interviewed 25 German patients with schizophrenia and found that almost half of them (49 %) experienced discrimination during social activities and exhibited perception of stigma. Chee *et al.* [11] studied 600 patients with mental illness at specialty and comprehensive hospitals in Singapore, almost half (48.6 %) of them felt that others looked down upon them, and slightly more than one third (37.1 %) of them felt being discriminated against in looking for jobs, and more than half (59.2 %) of the patients and 38.8 % believed that others would avoid him or her upon learning his or her condition. However, most of our understanding about internalized stigma in patients with schizophrenia comes from studies in Western countries.

Little evidence is available from China on internalized stigma and its risk factors. A recent hospital-based study of 441 patients with mental illness in China showed that stigma or lack of knowledge may hamper treatment for mental illness [12] In China, outpatients with schizophrenia account for more than 90 % of the total population with schizophrenia in China and constitute a particularly important group for study [13]. Patients with higher levels of education may have better understanding of their own illness by participating online support forums, reading articles about the traits of their illness such as easy recurrence, and the need for long term medication and prognosis, which may add on to the perception of stigma. On the other hand, patients with lower levels of education may lack such knowledge.. In the current exploratory cross-sectional study, we sought to assess the extent of internalized stigma among outpatients with schizophrenia and to investigate whether education level correlated with experience of stigma.

## Methods

### Patients

We carried out an exploratory cross-sectional-study of outpatients with schizophrenia who sought medical treatment at our hospital between January 2010 and January 2013. Subjects were screened for eligibility by reviewing the medical records of patients attending regular follow-up appointments. A patient was eligible for inclusion 1) if he or she was aged between 20 and 70 years of age; 2) if he or she met the diagnostic criteria for schizophrenia according to the International Classification of Diseases (ICD-10) [14]; 3) if he or she achieved remission after therapy with antipsychotic agents as defined by disappearance of mental symptoms, recover of insight, better social functioning and a brief psychosis rating scale (BPRS) score <30 [15]; 4) if he or she had no concurrent severe systemic diseases; 5) if there was no apparent abnormality in routine blood and urine chemistries, liver function or electrocardiogram (ECG). Individuals with clinically established impairment of insight, significant cognitive impairment or substance abuse in the previous three months were excluded.

### Ethical approval

The study protocol was approved by Tongde Hospital Ethics Committee and all study participants or their legal surrogates provided written informed consent.

### Patient evaluation

Patients were assessed for eligibility at a screening visit, with eligible patients returning for a baseline assessment in approximately one week, and then evaluated at subsequent follow-up visits. Patients were evaluated using the BPRS, the positive and negative syndrome scale (PANSS), the clinical global impression-severity of illness (CGI-SI) scale (1 point: very much improved; 7 points: very much worse) and the Stigma Scale for Mental Illness (SSMI 2C). The 27-item SSMI 2C is reported to have excellent internal consistency with an alpha of 0.91 and a test–retest correlation of 0.90 [16]. All three clinicians evaluating the patients were trained in using the SSMI 2C scale for this study with a kappa value of 0.91. It has 3 domains: domain I is the discrimination domain containing 12 items, which evaluates the degree of discrimination against the subjects due to the illness; domain II is the disclosure domain containing 9 items, which evaluates the degree of disclosure by the subjects; domain III is the positive aspects domain containing 6 items, which evaluates the intensity of perception of the disease by patients. The subscores from the three domains of SSMI 2C were added and higher scores indicate greater degree of stigma. The term “mental illness” was used throughout the questionnaire, but in the questionnaire respondents were encouraged to “think of it as whatever you feel is the best term for it.” Each item was rated on a 4-point Likert scale ranging from 1 = strongly disagree to 4 = strongly agree, with higher scores indicating higher internalized stigma. Evaluation was carried out by two senior psychiatrists and mean scores were reported.

All patients were evaluated by mental illness specialists who had been specifically trained in ICD-10 for this study with a kappa value of 0.85.

### Safety

In addition, vital signs and adverse events were monitored at baseline and post drug therapy using the treatment-emergent symptom scale (TESS) (NIMH, 1973). Safety assessments were based mainly on the occurrence, frequency, and severity of adverse events and were also based on comprehensive indexes, including physical examination, electrocardiography, and routine laboratory investigations. For all adverse events, where necessary, patients were withdrawn from the study.

### Statistical analysis

All results were expressed as mean ± SD and analyzed using the SPSS software version 15.0 (SPSS Inc., Chicago, IL). Student’s *t* test was used to compare BPRS, PANSS, TESS and CGI scores between two groups. Chi square test was used to compare differences in SSMI 2C scores and demographics. *P* < 0.05 was considered statistically significant.

## Results

### Demographic and disease characteristics of the study subjects

Two hundred thirty seven subjects were screened and 133 subjects were eligible for the study. Demographic and baseline characteristics of the study participants are shown in Table 1. The mean age of the study subjects was 32.05 ± 8.95 (range, 20 to 57) years and there were slightly fewer female patients (43.9 %) than male patients (54.1 %). Their mean course of illness was 4.32 ± 6.14 years (range, 1 months to 15 years). Their mean BPRS score was 19.87 ± 5.46, their mean PANSS score was 44.11 ± 13.1, and their mean CGI-SI score was 2.22 ± 0.81. In addition, their mean SSMI 2C score was 6.49 ± 0.9.

**Table 1:** Demographic and baseline characteristics of the study subjects (±S)

	All patients	High school or above	Middle school or less	*t’*	*P*
No. of patients	133	70	63		
Age, years					
Mean (SD)	32.05 ± 8.95	32.30 ± 8.67	31.78 ± 9.26	0.448 (υ = 129)	>0.05
Range	20–57	20–56	22–57		
Gender, n (%)					
Male	72 (54.1)	39 (55.7)	33 (52.4)	0.046 (*χ* ^2^)	>0.05
Drugs					>0.05
Atypical	123	64	59		
Typical	10	6	4		
Course of illness, years					
Mean (SD)	4.32 ± 6.14	4.07 ± 6.37	4.6 ± 5.87	0.298	>0.05
Range	0.08–15	0.17–13	0.08–15		
BPRS scores					
Mean (SD)	19.87 ± 5.46	19.34 ± 5.32	20.45 ± 5.61	1.17	>0.05
PANSS scores					
Mean (SD)	44.11 ± 13.1	44.06 ± 12.85	44.17 ± 13.38	0.071	>0.05
CGI-SI					
Mean (SD)	2.22 ± 0.81	2.13 ± 0.84	2.32 ± 0.78	0.73	>0.05
SSMI 2C					
Mean (SD)	6.49 ± 0.9	7.15 ± 0.98	5.75 ± 0.79	12.866	<0.05

We further analyzed the clinical records of patients according to levels of education. Patients who had received high school education or above and those who had received lower levels of education were comparable in demographic characteristics. They also had comparable BPRS score [high school or above, 19.34 ± 5.32 vs. middle school or below, 20.45 ± 5.61; *P* > 0.05], PANSS scale scores [high school or above, 44.06 ± 12.85 vs. middle school or below, 44.17 ± 13.38; *P* > 0.05], and CGI scores [high school or above, 2.13 ± 0.84 vs. middle school or below, 2.32 ± 0.78; *P* > 0.05] (Table 1).

Most patients (92.5 %, 123/133) took atypical drugs. The mean baseline TESS score was not statistically different compared to the mean post-therapy TESS score (*P* > 0.05). Common side effects are listed in Table 2. There was no statistical difference in the frequency of side effects between patients receiving high school education or above and those receiving middle school or below (*P* > 0.05).

**Table 2:** Side effects in the study participants

Variables	High school or above, *n* = 68 (n, %)	Middle school or below, *n* = 65 (n, %)	*P*
Extrapyrimidal side effects			
Muscle stiffness	3, 4.4	3, 4.6	>0.05
Tremor	1, 1.5	2, 3	>0.05
Twisting movement	1, 1.5	0, 0	>0.05
Immobility	2, 2.9	2, 3	>0.05
Cholinergic side effects			
Dry mouth	3, 4.4	5, 7.7	>0.05
Blurred vision	5, 7.4	6, 9.2	>0.05
Stuffy nose	4, 5.9	5, 7.7	>0.05
Salivation	3, 4.4	2, 3	>0.05
Constipation	4, 5.9	6, 9.2	>0.05

### Education and internalized stigma

As shown in Table 3, the mean SSMI 2C score of patients receiving high school education or above was 7.15 ± 0.98, which was markedly higher than that of patients receiving middle school education or below (5.75 ± 0.79) (*P* < 0.05). The mean domain I score of patients receiving high school education or above was 2.30 ± 0.76, which was comparable to that of patients receiving middle school education or below (2.07 ± 0.78, *P* > 0.05). The mean domain II score of patients receiving high school education or above was 2.42 ± 0.96, which was markedly higher than that of patients receiving middle school education or below (2.01 ± 0.79, *P* < 0.05). Moreover, the mean domain III score of patients receiving high school education or above was 2.43 ± 0.79, which was significantly higher than that of patients receiving middle school education or below (1.67 ± 0.77, *P* < 0.05).

**Table 3:** The Stigma Scale for Mental Illness (SSMI 2C) score stratified by education

	High school or above (*n* = 70)	Middle school or below (*n* = 63)	*P*
Discrimination factor	2.30 ± 0.76	2.07 ± 0.78	>0.05
1. I was discriminated against in education because of my mental illness.	2.11 ± 0.67	2.17 ± 0.58	
2. I sometimes felt discriminated against because of my mental illness.	2.25 ± 0.57	2.13 ± 0.69	
7 I was discriminated against by my superiors because of my mental illness.	1.84 ± 0.82	1.82 ± 0.75	
8. I was discriminated against by police because of my mental illness.	1.57 ± 0.76	1.69 ± 0.68	
10. I felt very lonely because of my mental illness.	2.28 ± 0.68	1.78 ± 0.79	
12. I would have had more opportunities if I had no mental illness.	2.87 ± 0.95	1.90 ± 0.82	
17. I felt angry at the attitude of others toward my mental illness.	2.64 ± 0.77	2.06 ± 0.74	
18. I encounter no trouble due to my mental illness.	2.27 ± 0.87	1.82 ± 0.78	
19 I felt discriminated against by medical staff due to my mental illness.	2.36 ± 0.82	2.86 ± 0.98	
20. People avoided me due to my mental illness.	2.74 ± 0.86	1.96 ± 0.79	
21. I was humiliated by others due to my mental illness.	2.30 ± 0.76	2.85 ± 0.93	
25. I feel life is unfair because of my mental illness.	2.58 ± 0.94	1.86 ± 0.79	
Concealment factor	2.42 ± 0.96	2.01 ± 0.79	<0.05
5. I am worried about telling others that I am on psychotherapy.	2.84 ± 0.87	1.88 ± 1.09	
11. I fear reaction by others if they find out I have mental illness.	2.43 ± 0.96	1.93 ± 0.78	
13. I do not care if my neighbors know about my mental illness.	1.33 ± 0.74	1.29 ± 0.67	
14. I will admit having mental illness if I am interviewed for a job.	0.71 ± 0.38	0.88 ± 0.49	
15. I fear telling others that I am on therapy for mental illness.	3.02 ± 0.69	2.67 ± 0.93	
16. I had to keep my mental illness secrete due to attitude of others.	2.73 ± 0.95	1.89 ± 0.72	
24. I do not like to tell others that I am having mental illness.	2.98 ± 1.87	2.53 ± 0.82	
26. I felt it was necessary to conceal my mental illness from my friends.	2.76 ± 0.77	2.89 ± 0.79	
27. I found it hard to tell others about my mental illness	3.04 ± 0.87	2.11 ± 0.84	
Positive effect factors	2.43 ± 0.79	1.67 ± 0.77	<0.05
3. I have become more considerate because of my mental illness.	2.52 ± 0.70	1.52 ± 0.73	
4 I do not feel terrible because of my mental illness.	2.22 ± 0.94	1.86 ± 0.89	
6. People understand my mental illness.	2.01 ± 0.84	1.77 ± 0.79	
9. I have become more tolerant of others because of my illness.	2.63 ± 0.76	1.22 ± 0.64	
22. I have become a stronger person because of my illness.	2.93 ± 0.76	2.06 ± 0.72	
23. I do not feel ashamed of my mental illness	2.31 ± 0.73	1.57 ± 0.83	

## Discussion

Our study revealed no marked difference in demographics, BPRS scores, PANSS scale scores and CGI scale scores and TESS scores among patients with different education backgrounds during convalescence. The side effects were mild and less frequent in our patients, which may be related to our use of atypical anti-psychotic drugs with benign safety profile, thus avoiding aggravation of internalized stigma of patients with schizophrenia due to side effects of anti-psychotic drugs [17]. We used SSMI 2C to determine internalized stigma of patients with schizophrenia with different educational backgrounds. We found no significant difference in discrimination domain scores among patients with different education backgrounds, suggesting that, regardless of educational background, patients with schizophrenia are reluctant to reveal their mental illness to others.

Internalized stigma leads to self-devaluation, shame and social withdrawal, rendering it difficult to overcome barriers to establish relationships and seek employment, seriously hindering the recovery process. It is important to help patients to cope with internalized stigma and to build up individual resistance in order to improve their well-being. The first step towards this goal is an understanding of internalized stigma in patients with schizophrenia. However, there is scant data on the presence of stigma in Chinese patients with schizophrenia. In the current study, we surveyed the presence of stigma in Chinese outpatients with schizophrenia and found that internalized stigma was pervasive in our study subjects. We further found that educational level was an important factor on perception of stigma by patients with schizophrenia: those who had high school education or above exhibited markedly higher SSMI 2C scores than those with middle school education or below.

With advances in the treatment of schizophrenia, psychiatrists increasingly pay attention to the management of mental health of psychiatric patients. It is well established that discrimination and stigma pose great barrier to the recovery of patients with schizophrenia [18], and stigma of patients with schizophrenia impacts on patient behavior in seeking medical therapy and also their adherence to therapy [7]. We have previously shown that stigma is pervasive in patients with schizophrenia during convalescence [19], and there is difference in internalized stigma among urban and rural patients with schizophrenia. Here, we further showed that education level also contributes to differences in internalized stigma in patients with schizophrenia, suggesting that proper interventions should be undertaken to tackle internalized stigma of patients with schizophrenia, which will help planning for recovery programs for and improvement of patients with schizophrenia.

Our study demonstrated that these patients, despite their different educational backgrounds, all experienced discrimination or humiliation in life and felt that life was not fair, indicating development of intense stigma resistance in these patients. Furthermore, patients with schizophrenia of different educational backgrounds exhibited marked difference in concealment domain scores. Those who had received high school education or above were more likely to conceal their illness from others than those who had received lower levels of education. They were more likely to receive psychotherapy, but were also less likely to disclose to others that they were on psychotherapy or anti-psychotherapeutic drugs. In addition, they were more sensitive to attitudes by others. On the other hand, patients who had received high school education or above were more likely to receive psychological counseling than anti-psychotherapeutic drugs and were more tolerant of others. Patients who had received middle school or below were more likely to focus on somatic illness rather than mental issues, indicating it is important to educate these patients about mental health. Furthermore, patients who received high school education or above had markedly higher scores in positive affect domain scores than those who received lower levels of education. They were more receptive to others and did not feel terrible and more independent compared to patients who received middle school education or below.

Because of the pervasive nature of stigma in patients with schizophrenia [19], apart from active drug therapy for preventing stigma, psychological education and aggressive psychological intervention are important for eliminating or alleviating stigma of patients with schizophrenia [20, 21]. As patients with different education backgrounds differ in stigma traits, psychological intervention should be individualized. Our findings suggest that patients who had received high school education or above should be assisted in their early return to the society and assumption of their work and family role. Patients who had received middle school education or less should be helped with restoration of self-confidence and provided with mental health education.

We did not address the issue of insight and stigma in the exploratory study. The relation between insight and education and stigma remains subtle and yet important, and insight in schizophrenia requires specific evaluation such as with the Birchwood’s Psychosis Insight Scale (BPIS). We will consider addressing the issue of insight and education and their relation with stigma in future studies by using scales such as the BPIS. The current study is also limited by its cross-sectional nature and the size of the study cohort. In addition, this is a single center experience with most patients coming from within the same province. Furthermore, the findings from the current study need to be confirmed by prospective multicenter study involving a larger patient population with longer follow up period.

## Conclusion

Our exploratory cross-sectional-study of outpatients with schizophrenia demonstrates that education level impacts on the perception of stigma by patients with schizophrenia and more psycho-education should be done to improve patients’ knowledge about schizophrenia.

## Abbreviations

BPIS: Birchwood’s Psychosis Insight Scale.
BPRS: brief psychosis rating scale
CFI: Camberwell Family Interview
CGI-SI: clinical global impression-severity of illness
ECG: electrocardiogram
FSI: Family Stigma Interview
ICD: International Classification of Diseases
PANSS: positive and negative syndrome scale
SSMI: Stigma Scale for Mental Illness
TESS: treatment-emergent symptom scale
